# Airborne particulate matter PM_2.5 _from Mexico City affects the generation of reactive oxygen species by blood neutrophils from asthmatics: an in vitro approach

**DOI:** 10.1186/1745-6673-4-17

**Published:** 2009-06-29

**Authors:** Martha Patricia Sierra-Vargas, Alberto Martin Guzman-Grenfell, Salvador Blanco-Jimenez, Jose David Sepulveda-Sanchez, Rosa Maria Bernabe-Cabanillas, Beatriz Cardenas-Gonzalez, Guillermo Ceballos, Juan Jose Hicks

**Affiliations:** 1 Departamento de Investigacion en Bioquimica y Medicina Ambiental, Instituto Nacional de Enfermedades Respiratorias, Ismael Cosio Villegas, Secretaria de Salud, Mexico; 2 Direccion de Investigacion Experimental en Contaminacion Atmosferica, Centro Nacional de Investigacion y Capacitacion Ambiental, Instituto Nacional de Ecologia, Mexico; 3 Universidad Autonoma Metropolitana, Unidad Iztapalapa, 09340, Mexico; 4 Laboratorio Interdisciplinario Seccion de Postgrado e Investigacion, Escuela Superior de Medicina, Instituto Politecnico Nacional, DF, Mexico

## Abstract

**Background:**

The Mexico City Metropolitan Area is densely populated, and toxic air pollutants are generated and concentrated at a higher rate because of its geographic characteristics. It is well known that exposure to particulate matter, especially to fine and ultra-fine particles, enhances the risk of cardio-respiratory diseases, especially in populations susceptible to oxidative stress. The aim of this study was to evaluate the effect of fine particles on the respiratory burst of circulating neutrophils from asthmatic patients living in Mexico City.

**Methods:**

In total, 6 subjects diagnosed with mild asthma and 11 healthy volunteers were asked to participate. Neutrophils were isolated from peripheral venous blood and incubated with fine particles, and the generation of reactive oxygen species was recorded by chemiluminescence. We also measured plasma lipoperoxidation susceptibility and plasma myeloperoxidase and paraoxonase activities by spectrophotometry.

**Results:**

Asthmatic patients showed significantly lower plasma paraoxonase activity, higher susceptibility to plasma lipoperoxidation and an increase in myeloperoxidase activity that differed significantly from the control group. In the presence of fine particles, neutrophils from asthmatic patients showed an increased tendency to generate reactive oxygen species after stimulation with fine particles (PM_2.5_).

**Conclusion:**

These findings suggest that asthmatic patients have higher oxidation of plasmatic lipids due to reduced antioxidant defense. Furthermore, fine particles tended to increase the respiratory burst of blood human neutrophils from the asthmatic group.

On the whole, increased myeloperoxidase activity and susceptibility to lipoperoxidation with a concomitant decrease in paraoxonase activity in asthmatic patients could favor lung infection and hence disrupt the control of asthmatic crises.

## Background

Air pollutants such as particulates and exhaust gases can reach considerable levels in areas of heavy traffic or in towns near mountains that form closed valleys where air movement is restricted, significantly increasing the toxic pollutant concentration. The Mexico City Metropolitan Area (MCMA) is one of the most densely populated cities in the world with 18 million inhabitants according to the 2000 census [1]. MCMA is an elevated basin approximately 2240 meters above sea level, surrounded by mountains to the south, west and east. At this altitude, 23% less oxygen is available than at sea level, which makes combustion less efficient [2]. In view of the diurnal cycle and city size, the distribution of nitrates suggests local photochemical production. On the other hand, sulfates appear to be produced on a regional scale. There are indications of new particle formation and growth events when sulfur dioxide (SO_2_) concentrations are high. The average atmospheric lifetime of sulfur emitted in Mexico City is 5.5 days, which is longer than the average lifetime of sulfur released in the rest of the world (3.9 days) [3]. Because of the altitude and the subtropical latitude of the Mexico City basin, the region receives intense solar radiation that promotes the efficient photochemical formation of pollutants. This changes their chemical composition during air transportation and results in particulate materials with different chemical properties.

For example, in the southeast zone of the city (Iztapalapa), the organic fraction of fine particles (PM_2.5_) at the Centro Nacional de Investigación y Capacitación Ambiental (National Center for Environmental Research and Training, CENICA) site is estimated to represent an average of 54.6% of the total mass, with the rest consisting of inorganic compounds (mainly ammonium nitrate and sulfate/ammonium salts), black carbon (BC) and soil [4]. Since air pollution seems to be associated with respiratory and cardiac diseases, particularly in children and older people, it is likely that the particles exacerbate pre-existing diseases in susceptible populations. Acute effects occur at relatively low pollutant concentrations and are associated with particles of apparently innocuous composition (largely carbon, ammonium sulfate and nitrate) [5]. Ultra-fine particles are contained in the fine fraction and the soluble material may translocate to extrapulmonary sites [6,7] for local cellular activation. This can increase the respiratory burst and concomitant generation of reactive oxygen species (ROS), chemical mediators and enzymes in peripheral cells, mainly neutrophils. It has been shown that activation of phagocytes both in vitro and in vivo can result in the generation of several ROS, including superoxide anion (O_2_^.-^) and hydrogen peroxide (H_2_O_2_), as well as the release of the heme enzyme myeloperoxidase (MPO) [8]. The increased generation of ROS due to the respiratory burst promotes an imbalance between ROS production and antioxidant defense that leads to oxidative stress leading to modification of molecules and/or disruption of cellular structures and tissue injury [9]. Due to high MPO activity, the generation of hypochlorous acid (HOCl) and reactive nitrogen species (RNS) also increases, resulting in the oxidation of tyrosine and nitrite and subsequent formation of tyrosyl and nitrogen dioxide (^.^NO_2_) radicals, respectively; these reactive intermediates can initiate the oxidation of lipids in the plasma membrane [10]. Another potentially important consequence of MPO activity is the consumption of nitric oxide and induction of endothelial dysfunction [8].

Although there is evidence that particulate air pollution has declined over time, epidemiological studies continue to show adverse health effects even at relatively low pollutant concentrations [11]. It is therefore likely that the increased air pollution and geographical characteristics of Mexico City have a significant impact on the health of the inhabitants [12,13].

In view of the mechanisms that have previously been proposed for health effects of pollution, we considered a parallel mechanism involving circulating neutrophils in addition to alveolar macrophages. Because neutrophils can migrate to the lung during acute inflammation or when macrophage phagocytosis is overwhelmed by the number of particles or invading microorganisms [14], the purposes of the present work were (i) to determine plasma paraoxonase (PON) and myeloperoxidase (MPO) activities, (ii) to evaluate the susceptibility of plasma circulating phospholipids to lipoperoxidation in a group of asthmatic patients compared to healthy volunteers and (iii) to measure in vitro ROS generation by peripheral human neutrophils obtained from healthy volunteers (HV) and asthmatic patients (AP) in contact with PM_2.5 _collected from MCMA.

## Methods

All reagents used in this study were from Sigma Chemical Co., St. Louis, MO, unless otherwise stated.

### Collection of particulate matter

Respirable particles [aerodynamic diameter < 10 μm (PM_10_)] and fine particles [< 2.5 μm (PM_2.5_)] were collected at the Centro Nacional de Investigación y Capacitación Ambiental (National Center for Environmental Research and Training, CENICA). Fourteen (PM_10_) and 13 (PM_2.5_) samples were obtained simultaneously over a 24 hour period, form May, 2005 to February, 2006. The samples were obtained with Andersen-Graseby high volume samplers onto quartz fiber filters (Whatman). The CENICA site is situated in southeast Mexico City (Iztapalapa zone) at the Autonomous Metropolitan University campus. It is the most populated area of the city with some food industries and is less than 2 km from the most important food merchandise distribution center in the city. The samplers were located on the roof of a four-story building.

Before and after sample collection, the filters were conditioned at 22 ± 3°C and 40 ± 5% RH during a 24 hour period and weighed with an analytical balance (Sartorious, sensitivity 10^-4 ^grams). After weighing, a section of the PM_10 _filter was subjected to chemical analysis following the standard procedures of USA EPA (1996 and 1998) by inductively coupled plasma atomic emission spectroscopy (Perkin Elmer, 3300 DV), and atomic absorption spectroscopy (Varian, Spectra A-2). A subsample of the PM_10 _filters were analyzed by electron microscopy (JEOL, JSM-5900 LV) coupled with Energy Dispersive Spectrophotometer (Oxford) with X ray detector in order to know the size distribution and individual composition of the particles. The complete PM_2.5 _filter was swept with a powder puff, collected in a polyethylene vial. The amount of particles recovered using this technique ranged from 18 to 80 mg. Once collected, the PM_2.5 _were transferred to the Biochemistry and Environmental Medicine Department at the Instituto Nacional de Enfermedades Respiratorias (National Institute for Respiratory Diseases; INER).

### Patients

The baseline characteristics of all subjects are shown in Table 1. The susceptibility of lipids to oxidation was used to calculate the sample size. According to the mean comparison formula [15] with a standard deviation of 157.53 and a difference of 616, Z_α _of 95% and a Z_β _of 80%, we obtained a sample size of 2. In total, 6 patients with mild to moderate asthma (AP) who came to the outpatient clinic for asthma management, were medicated with a β_2_-agonist, and fulfilled the criteria of the Global Initiative for Asthma [16,17] were recruited; 11 healthy volunteers (HV) were also enrolled. All of the subjects had lived in Mexico City for at least 5 years and were asymptomatic at the time of the experiment; none were smokers. On the morning of the experiment, patients and healthy volunteers underwent a spirometry test, which was performed by an experienced technician using a SensorMedics 2200 testing system (Yorba Linda, CA). The highest FVC and FEV_1 _values were selected from a minimum of three FVC maneuvers. All subjects gave written informed consent, and the protocol was approved by the ethics committee of the institution (C-03-04).

**Table 1:** General characteristics of the healthy volunteers and asthmatic patients included in the study.

	**Control Group**	**Asthma Group**	***p *value**
**Gender (M/F)**	4/7	0/6	

**Age**	43.5 ± 6.3	49.4 ± 11.5	0.1422

**BMI**	26.3 ± 3.4	29.6 ± 2.2	0.0721

**FVC%**	95.0 ± 12.2	90.4 ± 18.2	0.5407

**FEV_1_%**	99.4 ± 12.3	83.6 ± 21.5	0.0702

**FEF_25–75_%**	112.9 ± 23.9	54.11 ± 23.2	0.0002

### Cell and plasma isolation

Blood samples (10 ml) from both healthy volunteers and asthmatic patients were obtained by venepuncture, and neutrophils (N) were isolated with a density gradient using Polymorphprep™ solution (Axis-Shield PoC AS, Oslo, Norway) [18]. Four layers were obtained (plasma, monocytes, neutrophils, isolation media and erythrocytes). We recovered the first and third layer in order to quantitate the oxidative damage. The neutrophils were washed twice with Krebs-Ringer phosphate buffer, pH 7.4, supplemented with 1 mg/ml glucose (KRPG). Between the washes, hypotonic shock was used to remove any remaining red blood cells from the white cell preparation. The cell pellet was resuspended in KRPG buffer at a final concentration of 1 × 10^6 ^cells/ml.

### Paraoxonase activity

Before the analysis of paraoxonase (PON) activity, plasma was preincubated with eserine at 0.66 mM for 10 min at room temperature to inhibit butyrylcholinesterase activity and prevent interference with the determination of PON activity, which was measured following the technique of Abbot *et al*. and expressed as nmol p-nitrophenol/mg APO-A [19].

### Myeloperoxidase activity

First, 10 μl of plasma from HV or AP patients were placed in separate polyethylene tubes in 800 μl of 0.05 M acetate buffer, pH 5.4, supplemented with 0.3 M sucrose, 10 μl of 1.4 mM tetramethylbenzidine dissolved in dimethyl sulfoxide and 100 μl of 3.0 mM hydrogen peroxide. After incubation at 37°C for 10 min, 10 μl of catalase (1300 U/ml) and 100 μl of 0.2 M acetic acid were added. The samples were stirred and then centrifuged at 3000 ×g for 5 min and the absorbance at 655 nm was measured [20]. The results are expressed as MPO units. One unit (U) was defined as the quantity of enzyme necessary to catalyze an increase of 0.1 in the absorbance at 655 nm and 25°C. The specific activity was expressed as U MPO/mg protein.

### Susceptibility of lipids to oxidation

Circulating plasma phospholipids, which are rich in unsaturated fatty acids, were examined for their resistance to a specific oxidative aggressor that generates thiobarbituric acid reactive substances (TBARS) [21]. In this case, we performed an in vitro evaluation of TBARS formation using Fenton's reaction as a hydroxyl radical (HO^.^) generator and evaluated how much TBARS could be formed acutely in the plasma of each subject. The procedure was as follows: 5 μl of plasma from asthmatic patients or healthy volunteers was placed in a glass-covered tube with 7.2 mM Tris buffer (pH 8.2) and the mixture was incubated at 37°C for 15 min in the presence of 5 μM H_2_O_2 _and 5 μM FeCl_2_. At the end of the incubation, 1 mL of thiobarbituric acid 0.375% in 0.2 N HCl was added to the incubation mixture, which was stirred and boiled for 15 min. When the sample reached ambient temperature, 0.5 ml of 0.2 M HCl was added, and the absorbance at 532 nm was measured. The values obtained were expressed as μM of TBARS. The 1,1,3,3-tetramethoxypropane 0.1 mM in sulfuric acid 1% was used as standard.

### Quantification of reactive oxygen species

To measure the amount of free radicals generated, a chemiluminescence (CL) assay was performed as described by Trush [22] using a luminescence counter (20/20 n Luminometer, Turner BioSystems, Sunnyvale, CA). Luminol (5-amino-2,3-dihydro-1,4-phthalazinedione) was initially dissolved in DMSO to a concentration of 25 mM. This solution was stored in the dark at 4°C. On the morning of the experiment, 2 μl of this solution were added to the sample to give a final concentration of 100 μM. The CL response was measured in a polyethylene vial in a reaction volume of 0.5 ml, with 25 μl of the 1 × 10^6 ^cells/ml suspension containing neutrophils from healthy volunteers (NHV) or asthmatic patients (NAP). We first recorded the neutrophil CL signal over 10 minutes. After this time, we made a new sample the same way but this time we added 10 μl (1 mg/0.5 ml KRP) of PM_2.5 _suspension and recorded the CL response over 10 minutes.

### Statistical analysis

Data are expressed as means ± standard deviation. Paired t-tests were run to compare two groups, and ANOVA with post hoc Bonferroni multiple comparison tests were used for intergroup comparisons. Differences were considered significant when p was < 0.05. Data analyses were performed using the GraphPad Prism software (version 5.0 for Windows; GraphPad Software Inc., La Jolla, CA).

## Results

### Clinical Characteristics of Subjects

The general and clinical characteristics of the healthy volunteers and asthmatic patients are shown in Tables 1 and 2. All patients were in stable condition at the time of the study. An important point is that some clinical laboratory analyses showed significant differences between asthmatics and healthy volunteers; nevertheless, the measured parameters were not outside the limits established by institutional laboratory standard values.

**Table 2:** Biochemical characteristics of peripheral blood from the healthy volunteers and asthmatic patients.

	**Healthy volunteers**	**Asthmatic Patients**	***p *value**
**Eosinophils **(10^3^/mm^3^)	0.13 ± 0.04	0.42 ± 0.17	< 0.0001

**Neutrophils **(10^3^/mm^3^)	3.11 ± 0.55	3.84 ± 0.74	0.0364

**APO-A **(mg/dL)	133.3 ± 19.93	165.0 ± 27.59	0.0150

**MPO **(U/mg)	24.17 ± 18.21	52.58 ± 25.44	0.0250

**PON **(nmol/mg APO-A)	0.07 ± 0.02	0.02 ± 0.02	0.0005

**TBARS **(μM)	157.6 ± 115.4	497.6 ± 234.3	0.0008

Values are expressed as mean ± standard deviation.

### Particle Characteristics

**Figure 1:**
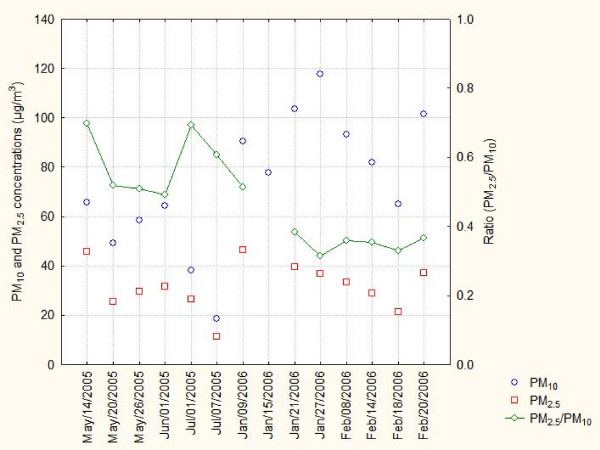
**Suspended particulate matter collected at the CENICA site**.

**Figure 2:**
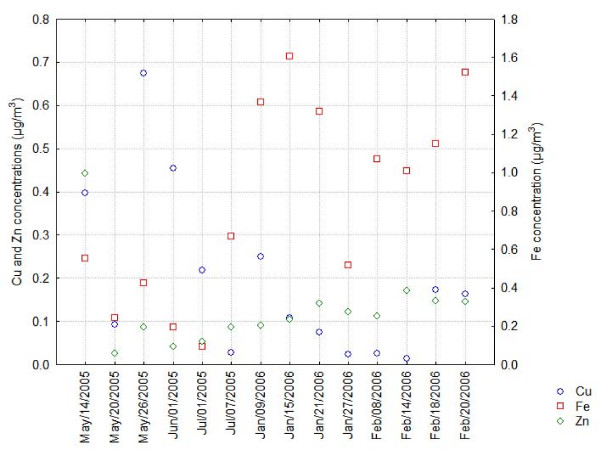
**Metallic composition of particulate matter (PM_10_) collected at the CENICA site**.

**Figure 3:**
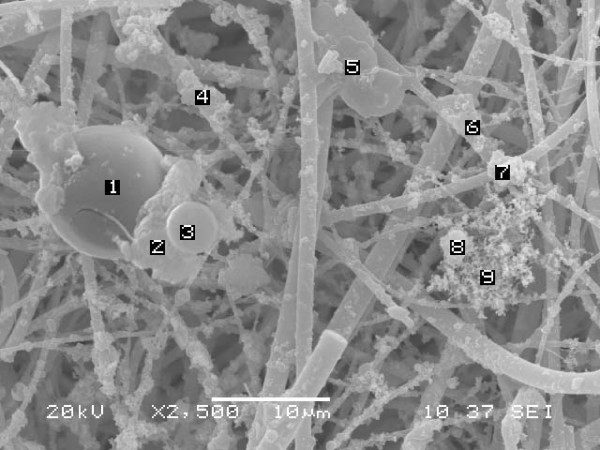
**Photomicrograph of respirable particles sampled at the CENICA site**. Numbers 1, 3 and 8 correspond to spheres; numbers 2, 4 and 7 correspond to clusters; 5 and 6 plates; number 9 corresponds to the reticular form. Numbers 1–5 correspond to the coarse fraction and numbers 6–9 to the fine fraction.

PM values measured at the CENICA site were 73 and 32 μg/m^3 ^for PM_10 _and PM_2.5_, respectively. The 24 hours average concentration measured in this study were below the Mexican air standars for PM_10 _(120 μg/m^3^) and PM_2.5 _(65 μg/m^3^), however the measured concentrations exceeded the Mexican annual standards of 50 μg/m^3 ^for PM_10 _and 15 μg/m^3 ^for PM_2.5 _campaign, showed seasonal variation, PM_2.5 _fraction accounted for 49 to 47% of the PM_10 _fraction during the rain season (May-June) and from 31 to 38% during the dry season (January-February) due to the effects of soil resuspension and land erosion which contributes to an increase on the PM_10 _fraction (Figure 1). Metals including Cu, Fe and Zn were evaluated in PM_10 _filter; the average concentrations found were 0.193, 0.838 and 0.127 μg/m^3^. A mass variability was found respecting those elements probably influenced by whether conditions and seasonal variation, eg. Fe mass as soil indicator, showed a two-fold increase during the dry season and correlated with PM_10 _concentration (p < 0.05); Zn and Cu were not clearly associated with each other, however on May 14^th^, an apparent Cu-Zn episode occurred. Zn showed a light increment during the dry season contrary to Cu concentration, Figure 2. In order to know the composition of PM_2.5_, samples of PM_10 _filters were analyzed by means of Scanning Electron Microscopy, 216 individual selected particles were manually evaluated using energy dispersive X-ray microanalysis (EDX). Individual shape and size particle characterization and semiquantitative percent composition of carbon, oxygen, S, Fe, and Cu were recorded in a database. Conformed information is presented in Table 3. The particles possessed diverse forms including spheres (1, 3 and 8), clusters (2, 4 and 7), plates (5 and 6) and reticular forms (9) corresponding to PM_10 _particles (indicated by numbers 1–5) and the fine fraction (6–9), (Figure 3). These analyses show that carbon and oxygen were the principal components, derived from incomplete combustion of fossil fuels and mineral contents; S only was observed in cluster (<4.1%) and irregular (<12%) forms in PM_10 _and in irregular forms in the fine fraction with less of 2% of its content. Moreover, the presence of metallic elements such as iron and copper was detected, the former reached the higher percent in cluster and irregular, both in the fine and PM_10 _fractions; the latter with exception of cluster shape in the fine fraction was found in all categories and accounted for less than 3% and 1.5% in the coarse and fine fractions, respectively. The presence of Fe and Cu content into spherical and soot aggregates of the fine fraction indicates a combination of natural and anthropogenic sources influenced by smelter and incineration emissions in the study area.

**Table 3:** SEM classification of individual PM_10_particles.

PM_10 _coarse fraction (diameter > 2.5 and < 10 μm)								
Spherical					Cluster		Irregular	
n = 13					n = 45		n = 86	

Element	Min		Max		Min	Max	Min	Max

C	26.5		68.6		17.2	60.0	14.2	59.1

O	25.6		45.0		25.7	44.8	11.9	49.4

S	nd		nd		0.8	4.1	3.8	12.0

Fe	0.4		1.9		0.3	12.0	0.4	11.8

Cu	0.4		1.0		0.7	1.5	0.4	3.6

PM_10 _fine fraction (diameter < 2.5 μm)								

Spherical			Cluster		Irregular		Soot Aggregate	
n = 12			n = 10		n = 28		n = 22	

Element	Min	Max	Min	Max	Min	Max	Min	Max

C	13.0	60.2	20.6	55.8	18.8	44.1	23.3	54.0

O	27.2	43.5	30.0	44.3	25.8	51.3	21.5	41.9

S	nd	nd	nd	nd	0.5	1.9	nd	nd

Fe	0.6	3.1	0.7	3.3	0.4	2.3	0.4	0.9

Cu	0.5	1.0	nd	nd	0.7	1.2	0.5	1.5

SEM = Scanning electron microscopy; nd = not detected

### In vitro Generation of ROS by Neutrophils

**Figure 4:**
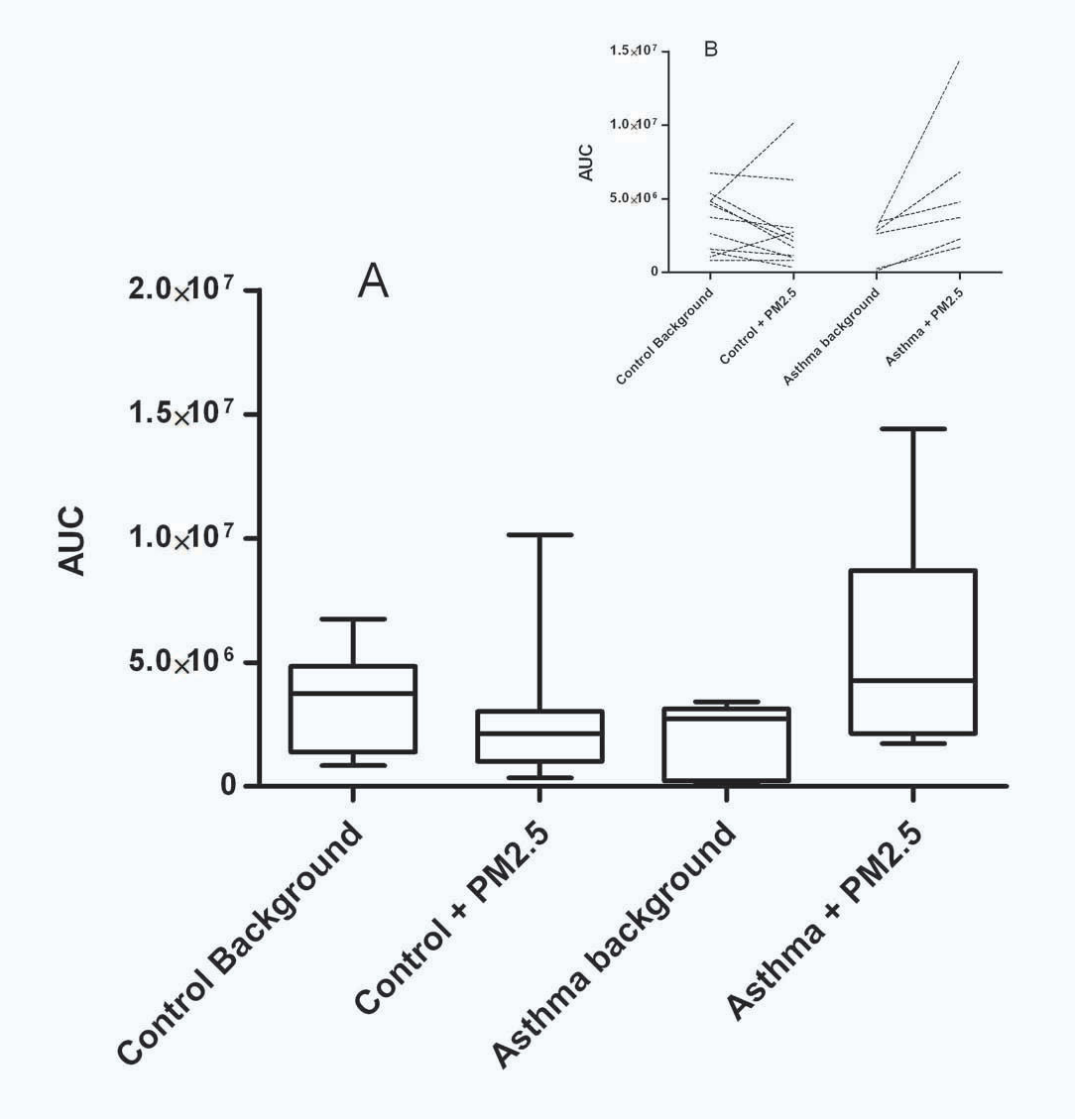
**In vitro generation of reactive oxygen and nitrogen species by neutrophils in contact with PM_2.5_**. A. In vitro production of reactive oxygen and nitrogen species by neutrophils from healthy volunteers (NHV) compared with neutrophils from asthmatic patients (NAP), measured by luminol-enhanced chemiluminescence and expressed as the area under the curve (AUC). The graph represents the mean of AUC for each group. B. Each line represents the chemiluminescence response of each subject that participated in the study, before and after treatment with PM_2.5_. The pattern shows a general increase in this response in the NAP group.

The in vitro generation of ROS was measured by luminol-enhanced chemiluminescence (CL) and expressed as the area under the curve (AUC). The CL AUC from NHV and NAP samples under basal conditions (background) were 3.425 × 10^6 ^± 2.018 × 10^6 ^and 2.044 × 10^6 ^± 1.462 × 10^6^, respectively, as a consequence of normal metabolism. The addition of PM_2.5 _did not stimulate CL in NHV (3.425 × 10^6 ^± 2.018 × 10^6 ^vs. 2.889 × 10^6 ^± 2.894 × 10^6^). In the NAP group, there was nearly a three-fold increase in the CL response; however, this increase failed to reach statistical significance, p = 0.07 (2.044 × 10^6 ^± 1.462 × 10^6 ^vs. 5.623 × 10^6 ^± 4.678 × 10^6^) (Figure 4A). When considering individual responses, the NHV group showed a decreased response after addition of PM_2.5 _when compared to the basal response (for example, one individual response was 1.148 × 10^6 ^vs. 0.157 × 10^6^) before and after particle addition, while the response in the NAP group after PM_2.5 _addition was higher (2.63 × 10^6 ^vs. 3.74 × 10^6^) (Figure 4B).

### Myeloperoxidase Activity in Plasma

Table 2 shows MPO activity expressed as units/mg protein (1 U = ΔA 0.01/min at 655 nm). Enzyme activity increased by 2.18-fold in the AP group when compared to the HV group (p < 0.05). In order to normalize the data, we took the ratio of MPO activity in the plasma to the chemiluminescence response since MPO is found in neutrophils; thus, we could account for the attenuation of the activation of neutrophils in the exposed and control groups (Figure 4).

### Paraoxonase Activity in Plasma

**Figure 5:**
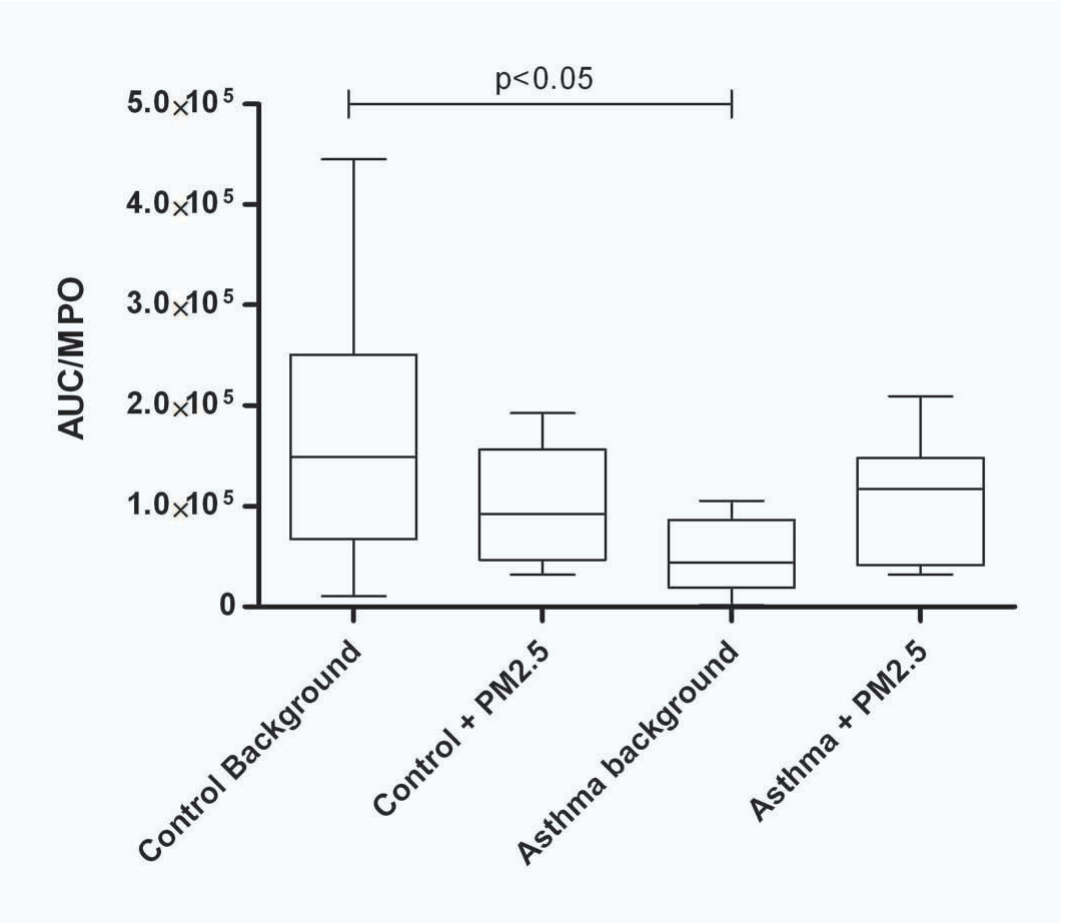
**Area under the curve/myeloperoxidase (AUC/MPO) activity ratio for asthmatic patients compared to healthy volunteers**. The ratio shows an increased inflammation response in cells exposed to PM_2.5_, in contrast to the decrease that is shown in the control group.

The plasma paraoxonase activity was expressed as nmol of p-nitrophenol phosphate formed per milligram of apolipoprotein A (Table 2). The paraoxonase activity was reduced by 3.5-fold when compared to the control group (p < 0.001). We normalized paraoxonase activity as described above for MPO activity (Figure 5).

### Susceptibility of Lipids to Oxidation

**Figure 6:**
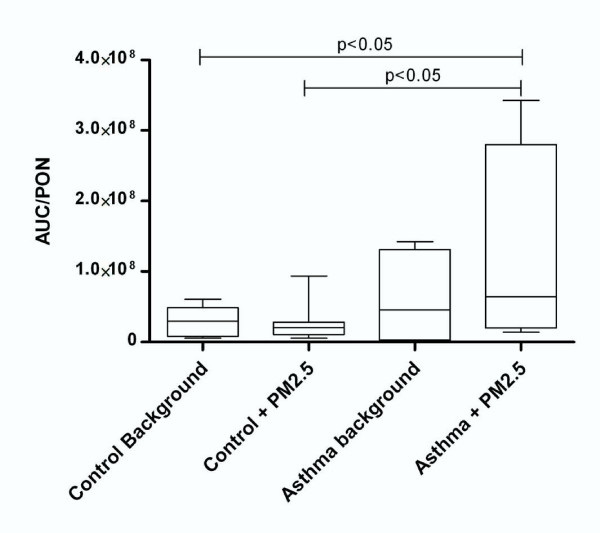
**Area under the curve/paraoxonase (AUC/PON) activity ratio for asthmatic patients compared to healthy volunteers**. The graph displays reactive oxygen species (ROS) generation as a function of enzyme protection, which is altered in the asthma group.

**Figure 7:**
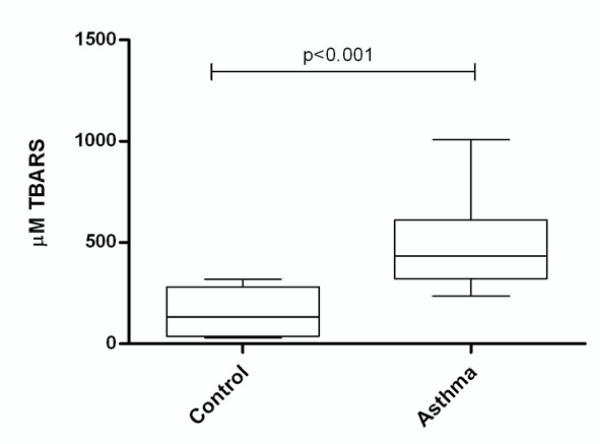
**Susceptibility of lipids to oxidation**. The graph shows a higher susceptibility of lipids from the asthmatic group to damage as a consequence of oxidative stress.

Table 2 also shows the in vitro formation of TBARS as a result of plasma lipoperoxidation by Fenton's reaction. TBARS formation was expressed as μmol per L of plasma (μM) and was 3-fold higher in the AP group than in the HV group (p < 0.001). Because the NAP response increased, we decided to compare it with the oxidative stress parameters in order to determine a general response. In Figure 5, the AUC/MPO ratio shows a pattern similar to that of the chemiluminescence signal. Reduced PON activity indicated inflammation generated by the loss of NAP modulation of ROS (Figure 6). This response is reflected as higher susceptibility to lipoperoxidation in those patients (Figure 7).

## Discussion

Oxidant generation is part of normal metabolism in many cell types and is critical for homeostasis. To protect against noxious oxidants, the lung has a well-developed antioxidant system [23] that includes a systemic response against air pollution. We previously demonstrated increased superoxide dismutase (SOD) activity and TBARS production during the first week of exposure to air pollutants in Mexico City among 21 volunteers who had never lived there [24]. Four months of exposure to air pollutants resulted in increased plasma antioxidant capacity that decreased lipoperoxidation, as measured by TBARS concentration [25]. An important factor for the mechanisms involved in cells death an injury, is the production of free radicals. Experimental and clinical data suggest that oxidants play a role in the pathogenesis of several respiratory disorders, including bronchial asthma [26]. In particular, increasing evidence shows that chronic airway inflammation typical of asthma results in increased oxidative stress in the airways. Moreover, many asthma triggers including viral infections and air pollutants may activate the production of ROS, thus resulting in inflammation in addition to the asthmatic symptoms [26].

The maintenance of basal ROS generation in response to the pollutant particles used to challenge neutrophils from healthy volunteers might be due to the efficient uptake of the particles by these cells, which rapidly engulf insoluble particles [27]. Although the response was not statistically significant, neutrophils from asthmatic patients showed an almost three-fold increase in in vitro ROS generation when exposed to PM_2.5_. This might be related to the activation of pro-inflammatory cytokines such as TNFα and IL-6 [28,29], which decreases the phagocytic and/or scavenger capacity [30,31] of neutrophils from these patients [27]. The exact mechanism by which particulate matter alters the phagocytic capacity is not fully understood and is a matter of great controversy. Some researchers have argued that this damage could be related to the cationic charge on the PM_2.5 _particles arising from the content of transition metals such as Fe and Cu [32-34]; other groups emphasize that organic and black carbon components found mainly in ultra-fine particles confer greater in vivo and in vitro toxicity than fine particles, and this effect is said to be independent of the soluble metal content [35]. The importance of charge in toxic xenobiotic molecules is related to the affinity of scavenger receptors for foreign material [36]; internalization seems to be increased in cells previously exposed to particulate matter. Furthermore, significantly increased MPO activity in plasma from asthmatics was observed when compared to the control group (Table 2). This may suggest an increased risk for development of asthmatic crises in these patients because of decreased bioavailability of nitric oxide. Otherwise, H_2_O_2 _is utilized by MPO [37] to generate reactive intermediates capable of initiating lipoperoxidation and protein damage through hypochlorite oxidation that generates reactive toxic aldehydes, increasing the likelihood of cellular injury [38]. In addition, asthmatic patients showed a significant decrease in paraoxonase activity; the presence of these markers is considered a risk factors for acute coronary syndromes [39-42]. Epidemiological, clinical and experimental evidence relates current levels of ambient air pollution to both respiratory and cardiovascular conditions. Oxidative stress, inflammation, induction of a pro-coagulatory state and dysfunction of the autonomic nervous system appear to play major roles [40]. Acute toxic effects resulting from ambient air pollution include changes in lung function, heart rate, blood pressure and an inflammatory state. The clinical consequences of such effects include respiratory symptoms, thrombosis, myocardial infarction, arrhythmia and stroke, all of which are related to acute oxidative stress caused by increased ROS and RNS, as well as inflammatory enzymes and other factors [43]. This suggests that some components of PM_2.5 _interact with membrane receptors, leading to activation of NADPH oxidase and increasing ROS generation in the NAP group. Unlike the NHV group, the NAP group was likely unable to counteract ROS generation due to asthma-mediated inflammation and concomitant oxidative stress, demonstrated by increased MPO activity and susceptibility to lipid oxidation, in addition to reduced PON activity. Collectively, the increased generation of ROS in these patients might be related to a concomitant decrease in nitric oxide bioavailability, thus increasing their susceptibility to asthmatic crises induced by air pollution.

## Conclusion

In summary, we observed a dual response in the generation of ROS and RNS by neutrophils from both asthmatic patients and healthy volunteers exposed to PM_2.5_. These findings suggest that PM_2.5 _pollutant materials affect blood neutrophils directly, inducing increased ROS and RNS generation in asthmatic patients. These individuals are unable to modulate this response due to their precarious oxidative stress condition, shown by increased MPO activity, reduced PON activity, and higher susceptibility to lipid oxidation, which can favor bacterial infection and increase the risk of asthmatic crises. Indeed, greater and more prolonged exposure to pollution is likely to induce more molecular damage in the exposed population; such damage includes the well-documented effects of oxidative stress, modification of circulating hormones and effects on their biological functions [44,45], abolished recognition of low density lipoprotein (LDL) receptors [46], cell damage and tissue injury. Further studies concerning the interactions of signaling pathways that specifically induce the release of different granule populations or bacterial internalization mechanisms of fine and ultra-fine particles may provide a better understanding about their toxicity.

## Abbreviations

NO_2_: Nitrogen dioxide; AP: Asthmatic patients; AUC: Area under the curve; BC: Black carbon; CENICA: National Center for Environmental Research and Training; CL: Chemiluminescence; Cu: Copper; DMSO: Dimethyl sulfoxide; Fe: Iron; FeCl_2_: Iron dichloride; FEV_1_: Forced expiratory volume in 1 second; FVC: Forced vital capacity; H_2_O_2_: Hydrogen peroxide; HCl: Hydrogen chloride; HO^.^: Hydroxyl radical; HOCl: Hypochlorous acid; HV: Healthy volunteers; IL-6: Interleukin-6; KRPG: Krebs-Ringer phosphate buffer supplemented with glucose; LDL: Lipoprotein; MCMA: Mexico City Metropolitan Area; MPO: Myeloperoxidase; N: Neutrophils; NADPH: Nicotinamide adenine dinucleotide phosphate reduced; NAP: neutrophils from asthmatic patients; NHV: neutrophils from healthy volunteers; O_2_^.-^: Superoxide anion; PM_10_: Particulate matter with aerodynamic diameter < 10 μm; PM_2.5_: Particulate matter with aerodynamic diameter < 2.5 μm; PON: Paraoxonase; RNS: Reactive nitrogen species; ROS: Reactive oxygen species; S: Sulfur; SO_2_: Sulfur dioxide; SOD: Superoxide dismutase; TBARS: Thiobarbituric acid reactive substances; TNFα: Tumor necrosis factor-alpha; USA EPA: United States of America Environmental Protection Agency; Zn: Zinc.

## Competing interests

The authors declare that they have no competing interests.

## Authors' contributions

All authors contributed equally to this work. All authors have read and approved the final manuscript.
